# Infallible Test for the Distinction of Real from Seeming Death

**Published:** 1872-10

**Authors:** 


Infallible Test for the Distinction of Real from Seeming Death.—Dr. Hugo Magnus, Assistant Physician to the Clinic of Prof. Dr. Forster, of Breslau, Germany (*Virchow's Archiv*, Vol. lv, 1872), has lately furnished a test for the decision of the important question, whether a seemingly dead body is really dead or not, which characterizes itself alike by its simplicity as well as scientific accuracy. As long as life is not entirely extinct, circulation and respiration, the two principal actions of vegetative life, must continue at least in a minimum degree; life without them is an impossibility. Any manifestation of the existence of one of these actions, becoming apparent to our senses, must certainly be an evidence of the existence of life. Dr. Magnus, therefore, ligates a finger about the middle of the first or second phalanx. If life is extinct, no change will take place; if it still exists in any degree, the ligated member will assume the well-known bluish-red discoloration following venous hyperæmia. Where the epidermis of the fingers is too thick and hard, he recommends the toes or the flaps of the ears for the experiment. R.

